# The effects of inactivated SARS‐CoV‐2 vaccination and subsequent infection of pregnant mice on the behaviors of offspring

**DOI:** 10.1002/ame2.12261

**Published:** 2022-07-31

**Authors:** Kaili Lin, Meixuan Liu, Lu Sun, Hongwei Qiao, Shunyi Wang, Sidan Pan, Hanjun Fu, Jingzhu Wang, Qiang Wei, Hong Gao

**Affiliations:** ^1^ Institute of Laboratory Animal Science, Chinese Academy of Medical Sciences and Comparative Medicine Center Peking Union Medical College Beijing China

**Keywords:** offspring, spatial reference memory, spontaneous locomotor activity

## Abstract

The mass inoculation of severe acute respiratory syndrome coronavirus 2 (SARS‐CoV‐2) vaccines to induce herd immunity is one of the most effective measures we can deploy in the fight against coronavirus disease 2019 (COVID‐19). Pregnant women are prone to a higher risk of COVID‐19, and maternal infection is a risk factor for a range of neurological disorders leading to abnormal behavior in adulthood. However, there are limited clinical data to support whether vaccination or infection post‐immunization in pregnant women can affect the behavioral cognition of fetuses in adulthood. In this study, human angiotensin‐converting enzyme 2 pregnant mice (F0 generation) were immunized with CoronaVac and then infected with SARS‐CoV‐2. Subsequently, we analyzed the behavioral cognition of their adult offspring (F1 generation) using the open‐field test and Morris water maze test. The adult F1 generation did not exhibit any impairments in spontaneous locomotor activity or spatial reference memory.

## Graphical abstract

15–16‐week‐age female hACE2 mice, weight 26–30 g, were randomly divided into three groups: a control group (the M/M group, *n* = 5), a vaccination group (the M/V group, *n* = 5), and a vaccination/infection group (the M/CV group, *n* = 5). F0 generations in the M/V group and M/CV group were injected two times (on day 0 and 28) with inactivated SARS‐CoV‐2 vaccine via intramuscular inoculation in the hind legs; 50 μl of vaccine (with a dose of 100 μl) was separately injected into the right and left hind legs. The M/M group was given an equal volume of PBS. The M/CV group was intranasally infected with 100 50% tissue culture infectious dose (TCID_50_) SARS‐CoV‐2 per mouse on 36 days after the first vaccination (13 days post‐pregnancy). The F0 generations were mated and became pregnant on 23 days after the first vaccination. In the above groups, two F1 generation mice (10 week‐age, weight 22–28 g) were randomly selected from each litter (*n* = 10 in each group) and then subjected to an open‐field test and the Morris water maze test (effects on the growth and development of F1 generation reported in another article).

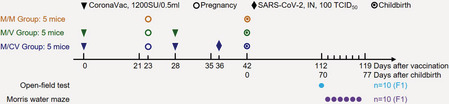

## 1 INTRODUCTION

The pandemic of pneumonia caused by severe acute respiratory syndrome coronavirus 2 (SARS‐CoV‐2) has caused great pressure and resulted in tragic consequences to the global public health and medical system. A massive Coronavirus Disease 2019 (COVID‐19) vaccination campaign is underway worldwide.^1^ Although pregnant women are prone to a higher risk of COVID‐19, clinical trials for the vaccines excluded pregnant and lactating women. Maternal infection may not only lead to premature delivery, abortion, and other adverse pregnancy outcomes but also increase the possibility of infection after childbirth.^2^, ^3^ Epidemiological studies have found that prenatal infection is a risk factor for a range of neurological disorders, accompanied by an increased risk of schizophrenia, working memory defects, and executive dysfunction.^4^ Maternal infection disrupts the immune balance between the maternal and fetal environments, leading to changes in the immune profile in the developing brain.^5^ The infection of pregnant women leads to the increase in cytokines such as interleukin‐6, which can pass through the placenta and act on placental cells to stimulate the production of downstream immune mediators in the uterus, and has become one of the risk factors for neurodevelopmental disorders in offspring.^6^

However, very limited data regarding the safety and validity of the vaccine during pregnancy are available. Consequently, women who are planning to become pregnant feel uneasy about being vaccinated. Our previous research results showed that the serum neutralizing antibody (NAb) and S1‐specific IgG titers of human angiotensin‐converting enzyme 2 (hACE2) mice inoculated with CoronaVac (an inactivated SARS‐CoV‐2 vaccine) were 256–512 and 6400–25 600 arbitrary units (AU) at 35 days after immunization.^7^, ^8^ The NAb titers of offspring in the vaccination group and the vaccination/infection group were 64–256 and 8–32 AU at weaning day, and the S1‐specific IgG titers were 1600–6400 and 800–3200 AU, indicating that the offspring successfully obtained NAb and IgG from the parental generation.^7^ In addition, there was no abnormality in the number, sex ratio, growth, and development of offspring in each group.^7^ In this research, hACE2 mice (F0 generation) were selected as a model to study the effects of immunization and post‐immunization infection in F0 generation on adult offspring (F1 generation) behaviors. According to the general principles for technical review of preclinical safety evaluation of preventive biological products in China, the inoculation dose of vaccine, in principle, should achieve the best immune response in animals. F0 generations were inoculated with a 100‐μl dose of CoronaVac at 0 and 28 days and infected with SARS‐CoV‐2 at 36 days.^9^ Then, we investigated the potential effects on spontaneous locomotor activity and spatial reference memory of the offspring. We intended to provide basic research data to support the clinical inoculation of pregnant women and women of childbearing age with inactivated SARS‐CoV‐2 vaccine.

## 2 METHODS

### 2.1 Animal grouping and processing methods

**FIGURE 1:**
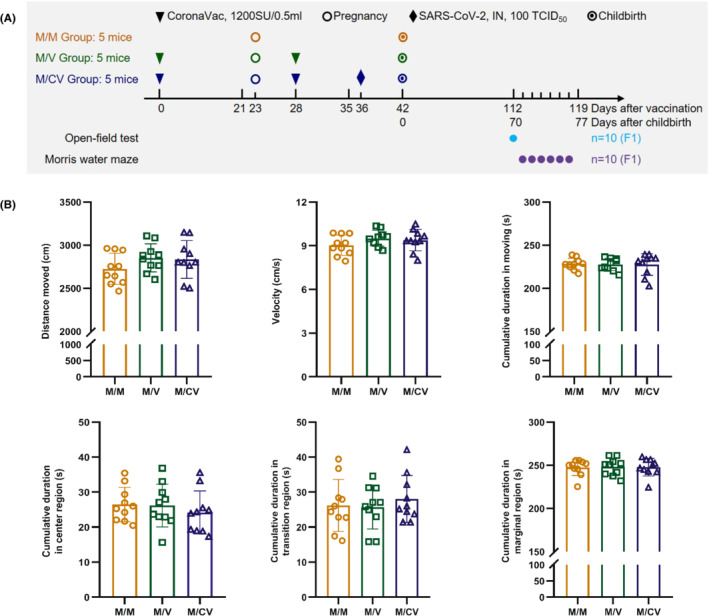
After the immunization of the F0 generations with inactivated SARS‐CoV‐2 vaccine and post‐immunization infection with SARS‐CoV‐2, the F1 generation (*n* = 10) exhibited no side effects on spontaneous locomotor activity. (A) Experimental design. F0 generation in the M/V and M/CV groups was injected twice (days 0 and 28) with inactivated SARS‐CoV‐2 vaccine via intramuscular injection in the hind legs; 50 μl of vaccine (100‐μl dose each time) was separately injected in the right and left hind legs. The M/M group was given an equal volume of PBS. The M/CV group was infected with 100 TCID_50_ SARS‐CoV‐2 intranasally at 36 days after the primary immunization (13 days post‐pregnancy). F0 generations were mated and became pregnant on day 23 after the first vaccination. (B) Movement distance, mean movement velocity, cumulative duration in moving, and cumulative duration in each region of the F1 generation during the open‐field test.

Female hACE2 mice (aged 15–16 weeks, weight: 26–30 g), provided by the Institute of Laboratory Animal Science (ILAS), Peking Union Medical College (PUMC), were randomly divided into three groups: a control group (the M/M group, *n* = 5), a vaccination group (the M/V group, *n* = 5), and a vaccination/infection group (the M/CV group, *n* = 5). F0 generations in the M/V group and M/CV group were injected twice (on days 0 and 28) with CoronaVac via intramuscular inoculation in the hind legs; 50 μl of vaccine (with a 100‐μl dose) was separately injected into the right and left hind legs. The M/M group was given an equal volume of phosphate buffered saline (PBS). The M/CV group was intranasally infected with 100 50% tissue culture infectious dose (TCID_50_) SARS‐CoV‐2 per mouse 36 days after the first vaccination (13 days post‐pregnancy). The F0 generations were mated and became pregnant 23 days after the first vaccination. In the aforementioned groups, two F1‐generation mice (aged 10 weeks, weight: 22–28 g, half male and half female) were randomly selected from each litter and then subjected to an open‐field test and the Morris water maze test (Figure 1A). The temperature was 23–25°C, and relative humidity was 55% ± 10%, with a 12‐h light–dark cycle. The F1 generations were transferred to the laboratory to adapt to the environment 30 min before the start of each behavioral experiment. All animal procedures were approved by ILAS, PUMC (reference: GH21001).

### 2.2 Viruses and vaccines

The SARS‐CoV‐2 virus designated as SARS‐CoV‐2/WH‐09/human/2020/CHN (GenBank: MT093631.2) was provided by ILAS, PUMC, China. CoronaVac (batch number: 20200411, labeling amount: 1200 SU/0.5 ml, specification 0.5 ml per dose) was produced by Sinovac Biotech Ltd, Beijing, China.

### 2.3 Open‐field test

For open‐field tests (50 cm per side), we considered a square region (20 cm per side) as the central region; this was centered at the center point of a dedicated open field. This was surrounded by a marginal region that was located 8 cm away from the margin. The transitional region is between the central region and the marginal region. The F1 generations were placed at the center point of the open field. We then recorded their spontaneous activities over a period of 5 min, including movement distance, mean movement velocity, movement time, and cumulative duration in each region, in the open field using a video‐based animal behavior tracking system.

### 2.4 Morris water maze test

The Morris water maze consisted of a cylinder without a lid (diameter, 120 cm; height, 40 cm); the water level was 25 cm; a platform was located 1.5 cm below the water. Then, a white odorless substance was added to the water maze and stirred evenly. Next, the pool was divided into four quadrants, the platform was placed in the northwest zone, and the water temperature was maintained at 22–25°C. A nontransparent curtain was labeled with markers, and data were recorded and analyzed with a Nolduc Etho Vision XT9 image acquisition and analysis system. Experiments were conducted over a 6‐day cycle; placement navigation was tested thrice a day between days 1 and 5. As the center of the quadrant arc was set as the delivery point, the F1 generations were placed randomly in the maze without repetition (Table 1). The experimental procedure lasted for 1 min. If the F1 generations could stand on the platform for 5 s, then platform‐seeking ability was considered to be successful; the time from water entry to successful platform localization was recorded as the latency. If the F1 generations were unable to stand on the platform for 1 min, then the latency would be recorded as 1 min. Cumulative movement distance was regarded as the latent movement distance. All of the mice were required to stand on the platform for 5 s regardless of whether they successfully sought out the platform. Space exploration experiments were carried out on day 6; the procedure lasted for 1 min, during which the platform was removed, and the mice were released from the southeast zone.

**TABLE 1:** Morris water maze spatial reference memory acquisition (place navigation test) experiment start quadrant

Days	First test	Second test	Third test
1	NE	SE	SW
2	SE	SW	NE
3	SW	NE	SE
4	NE	SE	SW
5	SE	SW	NE

Abbreviations: NE, northeast quadrant; NW, northwest quadrant (platform placement area); SE, southeast quadrant; SW, southwest quadrant.

### 2.5 Statistical analysis of data

Data analysis was carried out using GraphPad Prism 8.0 software. One‐way analysis of variance unpaired Student's *t*‐test was used to compare three groups. *p < 0.05 indicated statistically significant difference.

## 3 RESULTS

### 3.1 Inoculated with the inactivated SARS‐CoV‐2 vaccine and subsequent infection of F0 generation, the F1 generation exhibited no side effects on spontaneous locomotor activity

The open‐field test results revealed that the differences in cumulative movement distance, mean movement velocity, movement time, and each region's cumulative duration in the F1 generation were less prominent in the M/V group and the M/CV group when compared with those in the M/M group (*p* > 0.05) (Figure 1B). These data indicated that the offspring had no side effects on spontaneous locomotor activity during adulthood when the F0 generation had been immunized with CoronaVac and infected with SARS‐CoV‐2 after immunization.

### 3.2 Inoculated with the inactivated SARS‐CoV‐2 vaccine and subsequent infection of F0 generation, the F1 generation showed no adverse effects on the spatial reference memory

**FIGURE 2:**
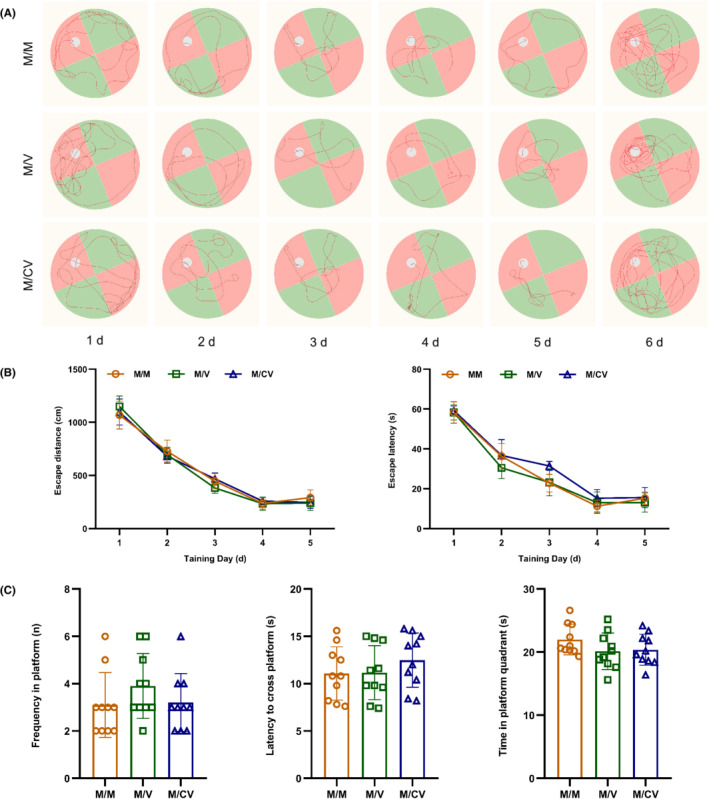
After the immunization of F0 generations with inactivated SARS‐CoV‐2 vaccine and post‐immunization infection with SARS‐CoV‐2, the F1 generation (*n* = 10) exhibited no effects with regard to spatial reference memory. (A) Motion trajectory of F1 generation during place navigation and spatial exploration in Morris water maze test; (B) latent movement distance and latency in the F1 generation during the Morris water maze test; and (C) platform crossing times, the latency of crossing the platform, and movement time in the platform quadrant of the F1 generation during the Morris water maze test.

According to data relating to place navigation in the Morris water maze, there were no significant differences in the latent movement distance and latency of the F1 generation when compared between the M/M, M/V, and M/CV groups (*p* > 0.05) (Figure 2B). Data relating to spatial exploration in the Morris water maze showed that there were no significant differences in the platform crossing time, the latency of first crossing the platform, and residence time in the platform quadrant for the F1 generation when compared between the M/M, M/V, and M/CV groups (*p* > 0.05) (Figure 2C). These results demonstrated that the immunization of F0 generation with the SARS‐CoV‐2 vaccine and post‐immunization infection with SARS‐CoV‐2 exerted no adverse effects on the spatial reference memory of F1 generation during adulthood.

## 4 DISCUSSION

Maternal exposure to microbial infection or immune activation during pregnancy has a long‐term impact on the development of the fetus after birth. Early research data show that the generation born during the pandemic of infectious diseases is at developmental risk.^10^ The infection of pregnant women with viruses, such as rubella, respiratory tract infection, influenza, and bacterial infection, is a risk factor for numerous neurological and psychiatric disorders in their offspring.^11^, ^12^, ^13^, ^14^ For a subset of women, maternal infection may be an inducement, leading to an altered trajectory of fetal brain development. Neuroimaging and behavioral studies have provided further evidence that schizophrenic patients exposed to infection in utero exhibited a characteristic pattern of brain volume changes, accompanied by an increased risk of schizophrenia, working memory defects, and executive dysfunction.^6^, ^15^ Furthermore, the dramatic increase in inflammatory cytokine levels in the fetus may have adverse effects on neurodevelopment.^15^, ^16^ Animal studies have shown that activation of the maternal immune system by viral infection (e.g., the influenza virus) may alter the levels of cytokines in the placenta, amniotic fluid, and fetal brain,^17^ thus affecting brain development and potentially inducing brain injury in the fetus. Pregnant rats were stimulated with immune activators, such as lipopolysaccharide, to produce strong immune responses, and the offspring exhibited similar changes in the brain and behavioral phenotypes with human disorders^18^; these events may eventually result in executive dysfunction as well as spatial learning and memory impairment.

Pregnancy vaccination can provide not only direct protection against infectious diseases, and its side effects, but also passive immunity for the newborn.^19^ Since December 2020, more than 185 000 pregnant women have been injected with COVID‐19 mRNA vaccine, and clinical data showed no increased risks of any adverse obstetric outcome, including preterm birth, stillbirth, neonatal death, and congenital malformations.^20^ In the global clinical trial of ChAdOx1 nCoV‐19, the pregnancy outcome analysis of 50 women who were pregnant after vaccination showed no effect on fertility rates and pregnancy outcomes by vaccination before pregnancy.^21^ The investigation of CoronaVac in pregnant women showed that two doses were 41% effective against symptomatic COVID‐19 and 85% effective against severe COVID‐19.^22^ Based on the clinical safety data of pregnant women vaccination, public health institutions globally have recommended COVID‐19 vaccination for pregnant women. Despite these recommendations, in many countries the proportion of pregnant women vaccinated remains low because there were no safety data on growth, development, and adult behaviors of offspring after vaccination of pregnant women. Therefore, we carried out a study on the behavior of offspring born by immunization and post‐immunization infection, aiming to provide reference for clinical vaccination of pregnant women.

The open‐field test is an established method that can be used to evaluate spontaneous locomotor activity and anxiety‐related behavior in animals and is implemented based on an animal's contradictory psychology of both fear and curiosity toward a new environment. For example, the cumulative time and frequency of animal behaviors reflect the spontaneous locomotor activity and exploratory behavior in the novel environment. Animals with high levels of anxiety predominantly move in the marginal region, with fewer activities in the central region. The Morris water maze, which does not require animals to be trained up front or restrict food and drinking water,^23^ is particularly used to assess the spatial learning and memory of animals and can be very useful for investigating the effects of drugs in rodents.^24^, ^25^ In this study, hACE2 pregnant mice were inoculated with the inactivated SARS‐CoV‐2 vaccine and infected with SARS‐CoV‐2 after immunization. We found that the offspring of these vaccinated animals had no side effect on spontaneous locomotor activity during adulthood and possessed good spatial learning and memory, thus implying that viral infection during pregnancy after inactivated SARS‐CoV‐2 vaccination did not increase the risk of spontaneous locomotor activity, learning, and memory in the offspring.

## AUTHOR CONTRIBUTIONS

Conceptualization, resources, methodology, and supervision: Hong Gao. Investigation: all authors. Writing the original draft: Kaili Lin. Review: Jingzhu Wang and Hanjun Fu. Writing, review, and editing: Qiang Wei and Hong Gao. Funding acquisition: Hong Gao.

## CONFLICT OF INTEREST

The authors declare that they have no conflicts of interest.

## ETHICS APPROVAL

All animal procedures were approved by the Institutional Animal Care and Use Committee at ILAS, PUMC (Reference: GH21001).
